# Supplementary material for: Are carbon emissions trading and green financial instruments synergistic? -Comprehensive quantitative research based on content analysis
Source: PLoS One. 2024 Mar 7;19(3):e0298601. doi: 10.1371/journal.pone.0298601 (PMC10919601; doi:10.1371/journal.pone.0298601)
Supplement: S1 Data — (ZIP) [file pone.0298601.s001.zip › Data/地级市Z_F工作报告.zip!/░▓╗╒/═¡┴Ω╩╨/└┤╫╘╣1⁄2╓┌║┼í╛┬φ┐╦╩2╛▌═°í┐_2011.docx]

各位代表：

现在，我代表市人民政府，向大会报告工作，请予审议，并请市政协委员和其他列席人员提出意见和建议。

一、2010年工作

刚刚过去的2010年，全市人民在省委、省政府和中共铜陵市委的坚强领导下，认真贯彻党的十七大和十七届三中、四中、五中全会精神，深入落实科学发展观，凝心聚力，积极进取，扎实工作，圆满完成了市十四届人大四次会议确定的各项目标和任务，国民经济呈现出增长加快、结构优化、后劲增强、民生改善的良好态势，社会大局保持和谐稳定。

预计，全年地区生产总值450亿元，增长17%，增速为“十一五”以来最好水平。财政收入88.6亿元，增长26.5%，在全省前移3个位次。社会消费品零售总额100亿元，增长19.3%。全社会固定资产投资360亿元，增长27.2%。新增就业2.14万人，城镇登记失业率3.9%。城镇居民人均可支配收入18700元，增长11.5%；农民人均纯收入6940元，增长12%。完成省政府下达的万元GDP能耗、二氧化硫和化学需氧量排放量控制指标。人口自然增长率2.17‰。

一年来，我们主要做了以下工作：

第一，着力抓好项目投资。我们继续大规模、高强度增加有效投入，完善项目谋划和推进机制，促进项目早开工、快建设。在建项目达到1000个，其中亿元以上项目130个，新开工项目570个。铜陵有色铜冶炼工艺技术升级改造、铜化集团大合成氨扩建、中发产业园、海亮产业园等一批投资额20亿元以上的重大项目相继开工建设；超远高密度印制电路板、华纳万吨铜箔等项目建成投产。争取国家和省各类转移支付资金20亿元，为历年之最。继续打造“千百亿工程”，加大工业强市等政策措施的落实力度，规模以上工业企业主营业务收入达到1200亿元，增长72%；经济效益综合指数提高80个百分点，再创历史新高。积极落实扩大消费政策，大力培育汽车、旅游休闲等消费热点，家电、汽车下乡和以旧换新产品人均销售额居全省前列。多措并举巩固外贸企稳回升势头，进出口总额增长40%，成为中国外贸百强城市。

第二，着力强化招商引资。我们抢抓承接产业转移机遇，快速启动“一区两片”规划建设。编制完成江南示范园起步区15平方公里规划。与省农垦集团合作组建了江北产业园开发公司。铜陵经济技术开发区扩区升级已上报国务院。继续加大招商引资力度，完善招商引资考核激励机制，开展“百名干部促招商”活动，先后组织和参加了中国（铜陵）投资贸易洽谈会、徽商大会、世博会安徽周和全国工商联、安徽省民营企业对接会等重大招商活动，与上海漕河泾开发区、深圳蛇口工业园建立了合作关系，与中建集团、中信集团等央企合作取得实质成果，成功引进中海阳光伏发电、上海蓝宝光电等一批亿元以上重大项目。全年实际利用外资突破3亿美元，利用市外内资260亿元。我市被评为2010年度苏商投资中国首选城市和最具投资潜力城市，承接产业转移示范市建设的品牌效应和抢滩效应加速显现，集群式产业转移和大规模整体承接势头明显。

第三，着力优化产业结构。我们把自主创新作为调整经济结构的中心环节，加快国家科技进步示范市和知识产权示范创建市建设，铜陵省级高新技术产业开发区、国家电子基础材料及新型元件高新技术产业化基地获批设立，中科院合肥技术转移中心铜陵分中心、合工大（铜陵）工程技术研究院组建成立，新增国家级高新技术企业24家，高新技术产业产值增长75%。完善了高层次人才项目经费资助、住房和生活补贴机制。加快培育先进装备制造、节能环保、新材料、新能源等战略性新兴产业。出台了关于大力发展服务业的实施意见，主城区中心商务区建设步伐加快。文化产业发展步入快车道，华谊兄弟等知名文化企业落户铜陵，青铜帝国等一批高端文化项目进展顺利。铜陵有色财务公司获准成立，铜源村镇银行挂牌开业，招商证券成功引进，“网络金融超市”在全省推广，金融机构各项存贷款余额双双突破400亿元。

第四，着力深化重点领域改革。我们在全省率先开展区直管社区综合管理体制改革试点，在铜陵县探索“大社会保障”体系新路子，在教育系统实行中小学教师系统内交流制度。全面推开公务用车货币化改革。全面完成基层医药卫生体制综合改革，实行基本药物“零差率”销售，政府投入2000万元购买公共卫生服务。基本完成经营服务性事业单位转企改制。全面完成文化行政管理、广播影视体制等七项文化体制改革任务。基本完成集体林权制度改革主要任务。加速培育各类市场主体，完善促进非公有制经济发展的政策措施，铜都阀门、洁雅科技等企业上市工作取得进展，新增民营企业1501户、个体工商户5300户。

第五，着力开展城乡大建设。我们积极推进新一轮城市总体规划修编工作。主城区畅通工程加快实施，淮河路等骨干路网拓宽改造相继完工，长江路地下通道即将建成，长江二路等工程开工建设。完成26个社区综合整治和一批环境景观工程。新增公交车80台，全面更新出租车。新城区建设步伐加快，西湖新区骨干路网基本建成，体育中心等重点工程动工兴建，东部和南部城区一批组团项目全面施工。综合交通体系建设积极推进，铜陵长江公铁两用大桥主桥墩已出水面，西湖互通立交、沿江高速铜陵连接线建成通车，城市外环线全线贯通。以乡镇村公共服务设施为重点的新农村建设全面推进，“千村百镇”示范工程深入实施。继续实施农业产业化“5132”提升工程，新增省级以上龙头企业7家，中华白姜文化园建成开园。现代林业示范市建设扎实推进。

第六，着力推进节能减排。我们重拳出击，通过启动节能预警调控、推动重点企业节能节电、加快淘汰落后产能等组合措施，在全省第一个完成阶段性节能调控目标，受到省政府表彰。强力推进污染减排，西湖污水处理厂、城市生活垃圾无害化处理等项目建成运行。全面启动国家环保模范城市创建工作，开展南部城区粉尘专项治理，集中整治运输泼洒和建筑施工扬尘，城区空气质量首次达到国家二级标准。出台了促进低碳经济发展的意见，滨江循环经济工业试验园和城山农业循环经济试验区分别完成投资18.5亿元和3亿元，循环经济发展示范效应逐步显现。

第七，着力提升民生工程。我们在省确定的33项民生工程基础上，组织实施了48项民生工程，年度目标全面完成，惠及95%以上的城乡居民，财政直接投入8亿多元，民生工程实际投入增长一倍以上。棚户区改造项目开工20个，竣工38万平方米，成为全省棚户区改造试点市。积极创建省级创业型城市，组织实施千人创业扶持计划，建成大学生创业园。在全省率先建立城镇居民社会养老保险制度，实现城乡养老保险全覆盖。新农合人均筹资标准提高到180元/年。在全省率先开展了“七位一体”专项救助，城乡救助体系进一步完善。城乡低保实现应保尽保，农村“五保户”供养水平全省领先。对困难弱势群体发放了临时价格补贴。抽样调查显示，城市居民主观评价幸福指数提高了4.3个百分点。

第八，着力加强社会建设。我们大力开展全国文明城市和国家卫生城市创建活动，国家卫生城市暗访考核顺利通过。成功举办了第十一届中国（铜陵）青铜文化博览会、省首届民俗文化节，国际铜雕艺术园首期建成开放。我市被省文化厅推荐为首批国家公共文化服务体系创建示范区，社区文化家园项目荣获文化部“群星奖”。在全省率先基本完成“校安工程”，高职、中职园区一期工程建成。城乡公共卫生服务体系进一步完善。第六次人口普查工作顺利开展。成为全国首批流动人口计划生育基本公共服务均等化试点市、人口计划生育综合改革示范市。全民健身运动广泛开展。全国残疾人工作示范市创建工作积极推进。与澳大利亚高嘉华市结成友好交流城市。妇女儿童、红十字、档案、地方志、民族、宗教、侨务、对台、外宣、消防、民防、防震减灾等各项事业都取得新进步。和谐社区建设经验在全省推广。实施重大事项社会稳定风险评估，进一步健全社会治安防控体系，成为全省社会管理创新试点市。构建“大调解”工作体系，深入开展“四大四进四送”活动。完善法律援助工作，高度重视产品质量和食品药品安全监管，安全生产形势总体平稳。

一年来，我们始终高度重视政府自身建设。扎实开展创先争优活动，提高科学发展的能力和水平。自觉接受人大法律监督和政协民主监督，密切与工商联、工会、共青团、妇联等人民团体的联系，认真听取各方面意见和建议，按时办结市人大代表建议148件、市政协提案308件。行政职权流程图绘制完成并逐项向社会公布，市直部门行政许可、审批事项基本进政务服务中心办理，政务公开深入推进，网络问政得到重视，依法行政和服务型政府建设有了新进展。严格贯彻落实《廉政准则》各项规定，强化重点领域、关键环节的行政监察和审计监督，坚决纠正损害群众利益的突出问题，政风建设取得新成效。

我们清醒地看到，前进道路上还存在不少问题。主要是：经济总量不大，三产服务业不强，人均生产总值较高与人均收入水平偏低、工业化率较高与工业化水平不高、城市化率较高与城市功能不完善的矛盾突出，节能减排压力和环境治理难度较大，土地、资金、人才等要素制约加剧。公共服务均等化水平不高，社会建设和管理仍须加强。同时，政府职能转变还不到位，作风不实等问题还在一定程度上存在。我们必须采取更加有力的措施加以解决，绝不辜负全市人民的期望和重托！

二、“十一五”回顾

“十一五”是我市经济社会发展中极不平凡的一个时期。面对诸多自然灾害和百年一遇金融危机，我们在省委、省政府和中共铜陵市委的坚强领导下，紧紧依靠全市人民，深入贯彻落实科学发展观，成功应对各种挑战和冲击，胜利完成了“十一五”规划各项目标任务，经济社会发展取得了令人鼓舞的成就，在提前八年全面建成小康社会、建设幸福铜陵的新征程上迈出了坚实步伐。

综合经济实力大步跨越。地区生产总值由“十五”末期的182.8亿元增加到450亿元，增长1.5倍。财政收入由24.5亿元增加到88.6亿元，增长2.6倍。完成固定资产投资1029亿元，超过“十一五”之前历年投资额的总和，一批事关全局的重大项目相继建成或开工建设。规模以上工业企业主营业务收入突破千亿元大关，实现工业增加值增长2.1倍。

转型发展迈出可喜步伐。积极推进全国资源型城市转型试点工作，棚户区改造、关闭矿山及改制企业职工养老和医疗保险等历史遗留问题逐步破解。铜产业支撑作用明显增强，化工产业向精细化工拓展，能源、水泥等传统产业由大到强，新材料、节能环保等战略性新兴产业加速发展。循环经济发展被国家发改委作为“铜陵模式”在全国推广，淘汰了一批落后产能，节能减排目标全面完成。开发各类新技术、新产品520余项，新增中国名牌4个、中国驰名商标3件，2010年高新技术产业产值是2005年的6倍。

城乡面貌发生巨大变化。新城区规划建设全面展开，“一主两副三城”的城市框架基本形成。拓宽改造城市主干道路253公里，新增市区道路总里程76公里。一批标志性城市工程和游园广场建成开放，水、电、气等城市基础设施得到整体改善。京台高速公路、沪渝高速公路和铜九铁路铜陵段建成通车，区内“六纵六横”干线路网基本建成，区域交通枢纽地位基本确立。新农村建设加快推进，实现村村通公路、通广播电视、通宽带、通客运班车，农村饮用水安全覆盖率居全省第一，23座水库除险加固工程全面完成，清淤整治农村河道500公里。都市型现代农业进一步发展，生姜、凤丹成为国家地理标志保护产品。

改革开放取得明显突破。在全省率先完成新一轮政府机构改革。国有企业改革积极推进，中小企业改制任务基本完成。农村综合改革不断深化。地方金融服务体系加快建立。非公经济快速发展。累计实际利用内、外资分别达到660亿元和10亿美元，是“十五”的11倍和4倍。全市开发园区建设面积达50平方公里，产业承接能力大大增强。

人民生活水平显著改善。累计投入财政资金21亿元，滚动实施民生工程53项，全市70万人得到实惠。新增就业10万人，城镇“零就业家庭”实现动态消除。在全省率先免除了洲圩区防洪排涝水电费，在全国率先实行涉农行政“零收费”。全民养老保险和全民医疗保障制度框架基本形成，五项社会保险参保水平不断提高。建立了更高水平的城乡困难群众大病医疗救助体系。城乡居民人均住房面积分别由23.8平方米和33.6平方米提高到26.9平方米和39平方米，低保家庭廉租住房实现应保尽保，多层次住房保障体系基本建立。城镇居民人均可支配收入由9524元增加到18700元；农民人均纯收入由3354元增加到6940元，连续4年高于城镇居民人均可支配收入增幅。

社会事业发展日益加快。义务教育阶段的中小学校、基层医疗卫生机构、文化和体育活动场所、计生服务站、乡镇司法事务所等标准化建设基本完成。铜陵学院、铜陵职业技术学院新校区建成启用。基础教育均衡发展走在全国前列，基本公共卫生服务均等化水平全省领先，社区建设处于全省一流水平，人口和计划生育工作保持全省先进。公共安全应急体系建设不断完善。信访维稳工作机制不断健全。社会治安综合防控体系不断强化，安保处置能力不断增强。军民共建工作扎实开展，国防动员、民兵预备役建设水平得到提高。群众性文明创建活动深入开展，城市软实力明显提升。基层民主法制建设进一步增强。社会大局保持和谐稳定，全市人民的幸福指数不断提高。

“十一五”的五年，是我市综合实力提升最快、城乡面貌变化最大、人民群众受惠最多的时期，是铜陵历史上发展最好最快的时期之一。五年来，我市还先后荣获全国创建文明城市工作先进城市、全国双拥模范城、全国社会治安综合治理优秀城市、全国科技进步示范市、中国优秀旅游城市、国家园林城市、金融生态市等称号，铜陵在国内外的知名度进一步提升。

五年的成绩令人振奋，经验弥足珍贵。我们始终坚持率先发展，在增加总量中优化结构，在调整升级中加快发展，经济社会发展一步一个脚印，一年一大台阶；我们始终坚持转型发展，着力推动观念转新、产业转型、体制转轨、环境转优，积极探索符合时代要求、具有铜陵特色的发展之路；我们始终坚持创新发展，把全民招商、开放承接与全民创业、培育内生动力紧密结合，城市化带动与城乡一体发展紧密结合，增强自主创新能力与促进可持续发展紧密结合，办成了一些打基础、管长远的大事；我们始终坚持和谐发展，突出保障和改善民生，努力解决人民群众最关心、最直接、最现实的利益问题；我们始终坚持聚力发展，提振精气神，埋头干事业，营造好环境，充分调动各方面的积极性和创造性，全市上下凝聚成干事创业的强大合力。

各位代表！“十一五”发展的辉煌成就，是科学发展观在铜都大地生动实践的结果，是省委、省政府和中共铜陵市委正确领导的结果，是全市上下团结拼搏、开拓奋进的结果！在此，我代表市人民政府，向全市各族人民，向广大工人、农民、知识分子、干部、驻铜解放军指战员、武警官兵和政法干警，向各民主党派、各人民团体和社会各界人士，致以崇高的敬意！向关心、支持我市现代化建设的中央和省属各部门、兄弟市和海内外友好人士，向在铜创业的境内外投资者和建设者，表示衷心的感谢！

三、“十二五”主要目标任务

未来五年，是我国工业化城镇化加速推进时期，是发展方式发生根本变革时期；是我市提前八年全面建成小康社会时期，是转型提升和建设幸福铜陵的加速期。我们面临大有可为的黄金发展期，肩负全市人民的新期待和更好更快发展的新使命。

根据中共铜陵市委《关于制定铜陵市国民经济和社会发展第十二个五年规划的建议》，市政府组织制定了《铜陵市国民经济和社会发展第十二个五年规划纲要（草案）》，提请本次大会审议。

总的指导思想是，全面贯彻党的十七大和十七届三中、四中、五中全会精神，以邓小平理论和“三个代表”重要思想为指导，深入贯彻落实科学发展观，紧紧围绕“转型跨越、富民强市、共建小康、同享幸福”这一主题，突出加快转变经济发展方式这条主线，坚持结构升级、创新强市、环境立市和城乡一体发展战略，着力深化改革开放，着力提升自主创新能力，切实推动观念转新、经济转型、体制转轨、环境转优，全面建成小康社会，推进幸福铜陵建设，着力开创又好又快发展新局面。

经济社会发展总体目标是：构筑世界铜都，提升皖中南中心城市地位，建设幸福铜陵。具体目标是：实现“一个提前、四个翻番、五个示范、两个率先”。即：到2012年，提前八年全面建成小康社会。到2015年，实现地区生产总值翻一番，达到1000亿元；财政收入翻一番，达到200亿元；固定资产投资翻一番，累计超过3000亿元；城乡居民收入翻一番，城镇居民人均可支配收入和农民人均纯收入分别达到38000元和15000元。努力打造转型发展示范市、承接产业转移示范市、循环经济发展示范市、创新发展示范市、城乡一体化发展示范市。率先实现基本公共服务均等化，率先基本实现教育现代化。

经济社会发展的主要任务是：

（一）经济建设方面

——在全省率先建成千亿铜产业和企业，成为具有全球竞争优势的世界铜产业基地，铜陵有色控股公司力争进入中国企业100强。成为全国重要的先进制造业基地。到2015年，实现规模以上工业企业主营业务收入2500亿元，新兴产业增加值占地区生产总值比重高于全省平均水平，形成特色鲜明的优势产业品牌集群。建成横港物流园、车站物流园等4个物流园区，培育10个以上年销售额超亿元的区域性商场和专业市场，成为皖中南现代物流中心、商贸中心和教育医疗等公共服务业高地，服务业增加值占地区生产总值的比重力争达35%。大力发展现代设施农业、优质高效农业、休闲观光农业，都市型现代农业发展迈上新台阶。

——高标准建成承接产业转移示范园区江南片核心区，铜陵经济技术开发区成为国家级开发区。五年实际利用内、外资分别达到2500亿元和22亿美元以上，引进世界500强和国内行业龙头企业10家以上，形成全方位、高水平、深层次的大开放格局。

——完善以企业为主体、市场为导向、产学研相结合的技术创新体系。到2015年，全社会科技研发投入占地区生产总值的比重达2.8%以上，高新技术产业增加值占规模以上工业增加值的比重达45%以上。每10万人拥有专利授权量超过200件。建设人才强市，实施“六大人才开发重点工程”，打造人才高地、创业福地。

（二）城乡建设方面

——围绕建成区100平方公里、满足100万人口生活居住的“双百”目标，按现代化大都市的标准建设城市，服务功能辐射周边地区，产业功能带动周边地区。将西湖新区打造成为主城区居住、商业、文体等功能的拓展区，将东部城区建设成为生态环境优美、功能设施齐全、现代气息浓厚的魅力新城，将南部城区建设成为商贸、物流、旅游等服务业为主的综合性新城区。

——建成城乡一体的公路交通网，实现过境通道高速化、区域干线快速化、城乡交通一体化、运输管理智能化、港口设施现代化。

——基本实现城乡基础设施一体化。高标准建设农村新型社区，实现安全饮用水、农村公交和城乡垃圾收运全覆盖，努力建设“美丽幸福乡村”。

（三）社会建设方面

——持续提升全面小康水平和幸福指数。五年新增城镇就业岗位12万个，城镇登记失业率控制在4%左右，创建国家级创业型城市。建立企业职工工资收入和离退休人员养老金合理增长的长效机制，促进收入分配公平，缩小城乡居民收入差距。建立社会保险、社会救助、社会福利、慈善事业相衔接，城乡广覆盖、功能更完善、标准再提高的社会保障体系。完成棚户区改造任务，实现住房保障全覆盖。

——推进城乡义务教育高位均衡发展，普及学前教育和高中阶段教育，基本建成皖中南职业教育基地。建立覆盖城乡居民的基本医疗卫生制度，实现人人享有基本医疗卫生服务。

——深化平安创建活动，把我市建设成为全省乃至全国最稳定的城市之一。

（四）生态文明建设方面

——构建比较完善的循环经济产业体系，将循环经济工业试验园、农业循环经济试验区建成国家级示范园区，努力实现低碳发展、绿色发展。

——全面淘汰落后产能。生活污水集中处理率达85%，生活垃圾无害化处理率达90%，矿山生态环境恢复治理率达50%，做到天更蓝、水更清、山更绿、城更美，力争成为国家环境保护模范城市，基本建成资源节约型、环境友好型社会。

（五）文化建设方面

——建成铜博物馆、文化馆、体育中心、会展中心、传媒大厦、图书新馆、美术馆、档案馆、气象科普公园等一批标志性文化体育设施，市、县、乡镇、社区文化场所达到国家一级建设标准，率先成为国家公共文化服务体系示范区。

——广泛开展群众性精神文明创建活动，群众的科学素养和文明素质明显提高。深化文化体制改革，构筑具有铜陵特色的“14322”文化产业体系，促进文化大发展大繁荣。

（六）改革方面

——全面完成国有企业改革，大力发展混合所有制经济，大中型企业全面建立现代企业制度。加快培育发展要素市场，形成统一开放、竞争有序的市场体系。优化非公经济发展环境，实施“万千百十”工程，到2015年非公经济占经济总量比重超过65%。

——建立充满活力的社会事业管理体制。扎实推进政府职能转变，全面提高政府效能，建设法治政府和服务政府。

各位代表！“十二五”美好蓝图令人憧憬、催人奋进。我们坚信，有74万铜都儿女敢于争先的优良传统、勇于拼搏的不屈意志，“十二五”宏伟目标一定会变成现实，铜陵这颗皖江明珠一定更加迷人，铜陵人民生活一定更加幸福美好！

四、2011年主要工作安排

今年，我们将迎来中国共产党诞生九十周年和市第九次党代会召开，开启“十二五”发展新局，做好今年的经济社会发展工作，意义重大。总的来看，发展环境较为有利。中央将继续实施积极的财政政策，陆续推出加快转变发展方式系列新政，转型跨越正逢其时；皖江城市带承接产业转移示范区、资源型城市转型、循环经济试点等政策叠加，发展机遇不可多得；一批新投产项目进入产能释放期，一批重大项目进入投资高峰期，增长态势更加强劲。但也要看到，外部发展环境具有不确定性，转型时期的矛盾日益凸显，各地发展的竞争十分激烈。我们一定要坚定发展信心，增强忧患意识，抓住一切机遇，积极开拓进取，求真求实，绝不懈怠，努力把改革发展稳定各项工作做得更好。

今年政府工作的总体要求是：高举中国特色社会主义伟大旗帜，全面贯彻党的十七大、十七届三中、四中、五中全会和中央经济工作会议精神，深入落实科学发展观，按照省委、省政府和市委的总体部署，突出“科学务实、赶超跨越、奋勇争先”的要求，以加快转变经济发展方式为主线，着力增加总量，优化结构，惠及民生，提升效能，经济发展保持全省第一方阵，社会建设争做全省排头，“五个示范市”建设开创新局面，为全面实施“十二五”规划开好局、起好步。

综合考虑各种因素，2011年全市经济社会发展主要预期目标是：地区生产总值突破500亿元，增长15%以上；财政收入突破100亿元，增长16%；全社会固定资产投资突破400亿元，同口径增长20%以上；社会消费品零售总额增长18%，居民消费价格指数不高于全省平均水平；城镇居民人均可支配收入突破2万元，农民人均纯收入力争达8000元，均增长13%以上；新增就业2.15万人，城镇登记失业率控制在4%以内；万元GDP能耗、二氧化硫、化学需氧量、氮氧化物和氨氮排放量均完成省控目标；人口自然增长率控制在6‰以内。

我们将重点做好以下八个方面工作：

（一）扩大有效投入，努力保持经济良好发展势头

持续加大投资和项目建设。统筹安排投资1000万元以上的重点项目超300个、亿元以上重点项目超165个、10亿元以上重点项目超29个。加快铜陵有色铜冶炼工艺技术升级改造、铜化集团大合成氨扩建等重大项目建设进度，确保皖能铜陵电厂100万千瓦机组、海螺1.2万吨水泥熟料生产线等重大项目早日建成投产。谋划和编制一批具有牵动能力、集聚效应和产业产品延伸的好项目、大项目，坚决摒弃“两高一低”项目。进一步完善项目工作机制，推动项目快落地、快建设、快投产。

加强经济运行调节。认真落实国家积极稳健、审慎灵活的宏观调控政策，梳理完善前几年我市出台的扶持企业发展的各项政策措施，进一步提高政策的针对性、灵活性和有效性，切实帮助企业减轻负担、抓好产销衔接和市场开拓。做好出口企业培育孵化工作，鼓励有条件的企业加快“走出去”。支持企业开展强强联合、技术改造、产品研发和品牌培育，推进工业化与信息化融合，增强核心竞争力。规模以上工业增加值增长20%以上。

切实做好保供应稳物价工作。抓好“菜篮子”工程，新增标准化蔬菜基地2000亩以上，改造完成幸福村、义安苑等菜市场，建设天桥菜市场。健全农副产品流通“绿色通道”，做好粮油、生猪等生活必需品的储备和投放工作。适时适度调控房地产市场，遏制房价过快上涨。加强价格监管，严厉打击串通涨价等不法行为。健全物价补贴联动机制，确保低收入群众基本生活不受影响。

（二）加速承接产业转移示范区建设，高水平推进招商引资

更大力度建设承接产业转移平台。推进铜陵经济技术开发区扩区升级，启动20平方公里基础设施建设，争取上半年成为国家级开发区。全面展开江南片承接产业转移示范园建设，力争基础设施投资超过10亿元。支持各县（区）省级开发区基础设施和产业配套能力建设。鼓励各园区建设特色产业园和异地产业园，研究出台共建共享利益分配机制，确保2—3个园中园项目开工建设，促进产业集群发展。

更大力度推进招商引资。坚持招商选资，围绕传统产业升级、新兴产业培育和循环经济补链等发展重点，编制项目投资指南。突出招大引强，瞄准世界500强企业、中央和省属国有大企业、与我市产业关联度高的行业龙头企业、全国知名民营企业，建立合作常态机制，重点引进一批产业层次高、投资规模大、带动能力强的大项目，每个县区及开发区都要引进2个以上投资规模在10亿元以上的重大项目。进一步营造全民大招商的氛围，完善招商引资考核奖惩办法，强化以商引商和产业招商，继续下大力气优化政务、市场和法制环境，努力让项目进得来、落得下。全年实际利用外资增长16%以上，利用内资增长25%以上。

更大力度拓宽融资渠道。大力引进异地金融机构来我市设立分支机构，新增小额贷款公司3-5家，创造条件设立社区银行。健全政银企合作长效机制，拓展“金融超市”服务功能，力争成功发行中小企业集合票据，探索推出商标权、专利权、订单质押贷款新品种，金融机构本外币贷款余额超500亿元。加大企业上市资源培育力度，上市公司再融资突破50亿元。规范政府融资平台，确保建投二期15亿企业债券发行成功。强化国家积极财政政策的对接和争取工作。

（三）聚焦产业结构调整，奋力推动经济转型升级

加快打造世界铜都。支持铜陵有色、精达特磁、全威铜业等企业加速向千百亿企业迈进。抓好铜工业园和铜加工节点项目建设，尽快形成铜加工产业集群。促进华纳国际等重点企业通过技术升级、产品延伸和产业配套，加快打造国家级铜基新材料产业基地。促进铜文化产业做大做强。加强铜产业技术开发和铜产品交易平台建设，鼓励企业加强标准化建设，抢占制高点。

培育发展战略性新兴产业。编制新兴产业培育专项规划，设立专项引导资金。支持锐展、中发、铜冠机械等重点企业加快汽车发动机及零部件、超高压变压器、井下无轨矿山专用设备等重点项目建设，做大特色装备制造产业；支持莱德、星辰等重点企业发展环境监测设备和LED照明、芯片、封装等产品，推动节能环保产业向规模化、高端化发展；支持中海阳等重点企业积极发展太阳能光伏产业链。新兴产业主营业务收入增长30%以上。

加速发展现代服务业。大力发展生产性服务业。抓紧规划建设江南示范园物流和铜拆解园、车站物流园，加快推进江北煤炭储配项目前期工作，构筑区域物流中心。积极培育总部经济、要素市场和中介等服务业。大力发展生活性服务业。以培育区域性专业市场和商贸综合体为重点，加快步行街特色商业街等重点项目建设，积极引进国际国内知名的品牌龙头企业，构筑区域商贸中心。加强目的地旅游城市建设。推动社区、家政等服务业发展。

（四）推进城乡大建设，着力提升皖中南中心城市地位

不断提升城市承载和辐射能力。完成新一轮城市总体规划和土地利用规划修编工作。配合做好京福高铁暨铜陵长江公铁两用大桥和南京至安庆城际铁路建设，促成铜南宣高速尽快复工。整合港口岸线资源，加强港口集装箱码头等基础设施建设，提高吞吐能力。加快构筑连接主城区和东、南部城区的快速交通网。统筹新、老城区的建设和开发，推进西湖新区主要路网及水电气配套设施与主城区贯通，加快西湖和车站两个片区整体开发，开工建设会展中心等项目，促进新区人口集聚，打造湖链城市新形象。建成东部和南部城区主干路网和一批组团项目。

加快实施畅通工程优化人居环境。积极推进北京路下穿老火车站等重点工程建设，完成俞家村立交桥等建设工程，综合整治城市支次道路，打通主城区的“丁字路”和“断头路”。坚持公交优先，优化公交线路，购置公交车辆150台，新增停车位1000个以上。完善城市功能布局，推进滨江生活岸线等综合整治，开工建设大铜官山公园和滨江大道二期景观绿化等工程，完成12个社区综合整治任务。创新城市管理模式，继续开展交通秩序、渣土泼洒等专项整治工作。

深入推进新农村建设。认真落实城乡一体发展“1+9”方案，以整村推进土地整治示范建设为抓手，加强乡村规划、管理和建设。实施乡村公路升级改造，开工建设顺安河航道整治二期工程，实施红星河综合治理，解决天门、顺安、钟鸣等乡镇边远地区的供水问题。继续实施新农村建设“千村百镇”示范工程、农村清洁工程，改善农村环境。

千方百计拓宽农民增收渠道。认真落实各项惠农政策，着力增加惠农直接补贴。提高农民的就业创业能力，增加工资性收入。加快都市型现代农业发展，积极扶持一批市级以上农产品加工龙头企业和农业休闲项目，促进农业增产增效。创新农村金融服务体系建设，拓展农村融资渠道。优化县区经济布局，构筑县区优势产业集群，提升县区经济发展水平。

（五）坚持深化改革，继续加快创新型城市建设

重点领域和关键环节改革要有新突破。推进市对辖区行政管理体制改革，实现重心下移、费随事转、责权统一。全面推行社区管理体制改革。巩固基层医药卫生体制综合改革成果，进一步深化公立医院改革。巩固文化体制改革成果。逐步完成事业单位分类改革。深入推进城乡一体化综合配套改革，在统一城乡户籍改革等方面争取突破。支持国有大企业移交办社会职能和主辅分离。加强国有产权转让和重大投资行为监管，提高资本运营效率，扩大财政预算试点范围，开展财政资金使用绩效评估。

自主创新能力要有新提高。推进国家铜深加工专利产业化试点基地等创新平台建设。支持企业创新能力建设，新增国家高新技术企业15家，组建省级以上研发机构5家。深入推进共性技术研发、科技创业孵化、科技资源共建共享等公共服务平台建设。加强知识产权保护。继续推进质量兴市，争创国家和省级名牌5个以上，促进重点行业实施技术标准发展战略。统筹各类人才队伍建设，加大急需的海内外高层次人才引进力度，开工建设人才公寓一期工程，争取完成技师学院筹建工作，建成城乡统一规范的人力资源市场。

全民创业要有新气象。以创建省级创业型城市为目标，以大学生、农民工等创业园区为载体，深入推进千人创业扶持计划，完善政策扶持、创业培训、创业服务“三位一体”的创业帮扶工作机制。加大扶优扶强力度，着力促进全市规模以上非公企业和22家扶优扶强民营企业加快发展，加快推动我市94家省级重点调度中小企业做大做强，力争有更多的企业进入省级“专精特新”中小企业行列。新增民营企业1500户，新增个体工商户5000户。

（六）突出“两型”社会建设，不断改善城乡环境质量

扎实抓好节能减排工作。坚持标本兼治，提高新上项目环境准入门槛，严格落实环保“三同时”制度。强化目标责任制，盯紧重点企业、重点行业、关键环节和薄弱地区，严格监控“三高”行业，确保完成节能减排年度目标任务。加快淘汰落后产能，完成石料矿山全面整治，促进煤矿企业早日退出转型。推动各企业开展清洁生产。开展扬尘污染全城区综合整治，城市空气中可吸入颗粒物日均值比上年下降10%。对污染较重的工业区居民逐步实施整体搬迁。加快主城区污水管网配套建设。继续推进狼尾湖综合整治、废弃矿山和尾砂库生态治理修复。全面掀起创建国家环保模范城市热潮。大力推进现代林业示范市建设，让森林进城、园林下乡，增加森林碳汇，实现有地见绿。

深入推进循环经济发展示范市建设。研究制定促进企业间循环利用的支持政策，推动产业上下延伸、企业梯级耦合，加快构筑有色、化工、建材等循环经济产业链。完善循环经济园区协调推进机制，加快滨江循环经济工业试验园热电联产等关键节点项目建设，落实横港循环经济工业示范区建设规划，加大城山农业循环经济试验区基础设施投入，促进各园区企业循环共生、绿色发展。加快铜陵有色铜冶炼烟气余热利用节能技术改造等19个循环经济项目建设。争取列为国家循环经济示范市。

（七）倾力改善民生，进一步提高人民群众幸福指数

拓展提升民生工程。坚持把改善民生作为政府工作的出发点和落脚点，更加注重增加城乡居民收入，更加注重促进社会公平公正，更加注重提高全市人民幸福指数。实施 48项民生工程，切实让全市人民得到更多实惠。

提高居民收入水平。坚持创业富民，以创业促就业，完善就业援助制度。健全企业工资集体协商制度，兑现事业单位绩效工资，增加居民工资性收入。依法保护劳动者和被征迁农民的合法权益，鼓励居民扩大财产性收入。组织实施城镇居民养老保险试点，扩大新型农村养老保险覆盖面，实施特大医疗费用统筹办法，将全市老工伤人员逐步纳入工伤保险基金管理。提高企业退休人员基本养老金标准。完善最低生活保障标准与物价波动的联动机制和科学调整的自然增长机制。高度关注城乡困难群众生活，研究因病致贫和多病多残人群救助办法。更加关注老龄事业和养老服务体系建设，加快发展社会福利和慈善事业。

加快保障性住房建设。加大棚户区改造推进力度，强化责任、落实资金，棚户区改造施工面积160万平方米。完善困难群体住房保障制度，启动公共租赁住房建设，廉租住房保障户数不低于7400户。加快安置房建设，兑现安置承诺。做好住房公积金归集、扩面和贷款管理工作。

提升教育均衡发展水平。着力提高教育质量，促进教育公平。完善扶贫助学机制，实现困难家庭学生资助全覆盖。通过政府购买服务解决农村中小学学生乘车安全问题。深入推进义务教育标准化学校建设，完善区域内教师和校长交流制度，促进优质教育资源城乡共享。新建和改造幼儿园11所，创建市级一类幼儿园7所。优化高中阶段办学布局，办好铜陵职业技术学院和中职教育中心，积极引进高等教育资源，促进各类教育均衡发展。

推进城乡医疗卫生服务均等化。完成新建社区和村改居社区卫生机构建设任务，建设居民电子健康档案，提高免费服务标准。全面落实国家免疫规划，抓好重大疾病和地方病防控，强化突发公共卫生事件处置。进一步提高医疗公共服务水平，市人民医院改扩建项目门诊大楼主体封顶，市二院康复病房楼、中医院新院区等投入使用。

突出加强文化建设。以创建国家公共文化服务体系示范区为契机，提高乡镇、村和社区公共文化服务设施建设使用水平，加快铜博物馆、文化馆等重点文化工程建设。加大文化创意、影视动漫等项目的引进力度，着力培育一批骨干文化企业，促进文化产业发展。大力开展群众性文化娱乐活动，积极发展新闻出版、广播影视事业，促进哲学社会科学、文化艺术繁荣发展。

协调推进各项社会事业。完善基层公共体育设施，促进体育事业和产业协调发展。组织开展全国流动人口计划生育基本公共服务均等化试点市和人口计划生育综合改革示范市工作。启动档案馆新馆建设。支持发展妇女儿童事业。继续加强外事、侨务、民族、宗教、对台、气象、防震减灾、地方志、老龄、红十字等工作。建成全国残疾人工作示范市。

（八）强化文明创建工作，切实促进社会和谐稳定

大力开展文明创建活动。深入推进全国文明城市创建工作。实现国家卫生城市创建目标。开展和谐社区示范创建活动，建设文明和谐幸福新家园。推进法治县区、民主法治村（社区）创建活动，扩大法律援助覆盖面。充分发挥工会、共青团、妇联等人民团体的桥梁纽带作用，规范发展各类社会组织。支持驻铜部队建设，做好国防动员、民兵预备役和人防工作。扎实推进全国双拥模范城创建工作，力争实现“三连冠”目标。

全力维护社会稳定。大力推进全省社会管理创新试点市建设，深入推进“平安铜陵”建设，强化社会治安综合治理，开展“打黑除恶”和打击“两抢一盗”专项斗争，加大治安复杂地区和突出治安问题专项整治。继续推进“四大四进四送”活动，健全矛盾纠纷排查化解机制，有效预防和妥善处置群体性事件，维护群众合法权益。强化落实安全生产责任制，深入排查和消除事故隐患，重点抓好煤矿、非煤矿山、危险化学品、道路交通、消防等领域的安全监管，坚决防止发生重特大事故。推进药品和餐饮服务食品安全示范县区建设。健全政府应急管理体系，提高保障公共安全和应对突发事件的能力，确保人民群众生命财产安全。

各位代表！为实现各项工作目标，必须努力建设人民满意的法治政府、服务政府。一要强化宗旨意识。各级政府公务员要切实做到从思想上尊重群众、感情上贴近群众、工作上依靠群众，始终与人民群众同呼吸、共命运、心连心。创新群众工作方式方法，健全群众工作制度机制，在解决群众所盼上雪中送炭，在帮助群众所需上不遗余力，在为群众谋更多福祉上尽心尽责。二要坚持依法行政。行政机关工作人员特别是领导干部要带头学法、尊法、守法、用法，自觉养成依法办事的习惯，切实提高运用法治思维和法律手段解决经济社会发展中突出矛盾和问题的能力。深入推进规范行政执法示范市建设，进一步规范和减少行政审批，落实行政处罚自由裁量权细化量化执行标准，全面推行权力运行公示制度。大力推进政府信息公开、办事公开，进一步完善财政预算、政府投资等信息发布制度。自觉接受人大法律监督和政协民主监督，认真办理人大议案、代表建议和政协提案，充分听取各民主党派、工商联、无党派人士及各人民团体的意见，广泛接受社会公众和新闻舆论监督。三要提升服务水平。积极探索开展审批权相对集中试点改革，适当下放与市民生产生活息息相关的行政审批权限，实现从审批监管向便民服务转变。继续完善政务服务中心功能，构建基本建设项目联合审批平台，继续推进全程代理、“一单联办”、“一表收费”等便民服务方式，开展政务服务标准化、规范化建设。四要提高工作效率。继续推行“一线工作法”，严格落实首问负责制、办事限时制等制度，做到有事即办，办就落实。深化“千项办件看效能”专项监察活动，实行行政审批项目电子集中监控，切实纠正工作中存在的推诿扯皮、敷衍塞责和消极拖拉等现象。加强电子政务建设，努力打造成本低、服务优、效率高的政务环境。五要做到廉洁从政。坚持把反腐倡廉建设放在更加突出的位置，严格落实党风廉政建设责任制和《廉政准则》，加大教育、监督、改革和制度创新力度。逐步建立覆盖各领域、延伸到各地各部门的廉政风险点防控管理体系。积极推进职务消费改革，制定出台公务接待管理办法。加强对国有资产、财政资金及各类政府建设项目的审计监督，开展工程建设、国土资源等领域突出问题的专项治理，严肃查处违法违纪行为，坚决纠正损害群众利益的不正之风。六要严格责任落实。完善目标管理和责任落实机制，严肃责任追究，做到令出必行、行必有果，切实提高政府的执行力和公信力。深入开展创先争优活动，各级政府工作人员必须始终保持昂扬向上的精神状态，始终保持干事创业的工作激情，解放思想，与时俱进，勤勉尽责，忠诚奉献，努力做出人民满意的实绩。

各位代表！科学发展的新征程已经开启。充满希望的铜都大地，一幅转型跨越、富民强市、共建小康、同享幸福的锦绣画卷正徐徐展开，发奋图强正当时，拼搏创业齐努力。让我们高举中国特色社会主义伟大旗帜，深入贯彻落实科学发展观，在中共铜陵市委的坚强领导下，万众一心，奋勇向前，为圆满完成今年各项目标任务，夺取提前八年全面建

成小康社会和建设幸福铜陵的新胜利，谱写“十二五”发展更加辉煌灿烂的新篇章而努力奋斗！
